# EEG-based frontal excitation/inhibition balance as an objective biomarker for cognitive fatigue across multiple sclerosis and Long COVID

**DOI:** 10.1017/S0033291725103024

**Published:** 2026-01-15

**Authors:** Stefanie Linnhoff, Roi Cohen Kadosh, Tino Zaehle

**Affiliations:** 1 Department of Neurology, Otto-von-Guericke University, Magdeburg, Germany; 2 Center for Behavioral Brain Sciences, Otto-von-Guericke University, Magdeburg, Germany; 3 School of Psychology, University of Surrey, Guildford, UK; 4 Institute for Medical Psychology, Otto-von-Guericke University, Magdeburg, Germany; 5 German Centre for Mental Health (DZPG), partner site Halle-Jena-Magdeburg, Magdeburg, Germany

**Keywords:** aperiodic exponent, excitation/inhibition balance, EEG biomarker, fatigue, Long COVID, multiple sclerosis

## Abstract

**Background:**

Cognitive fatigue is a prevalent and disabling symptom in neurological and post-viral conditions, including multiple sclerosis (MS) and Long COVID. Assessment relies largely on self-report, and no validated objective biomarker exists, limiting reliable diagnosis and treatment monitoring. The aperiodic exponent of the Electroencephalogram (EEG) power spectrum, reflecting the excitation/inhibition (E/I) balance, is a promising candidate biomarker. We examined whether aperiodic exponent values can objectively identify pathological fatigue and assessed their classification accuracy.

**Methods:**

We conducted a cross-sectional study, including 119 participants: 36 healthy controls, 33 with Long COVID-related fatigue (LCOF), and 50 with MS (23 fatigued and 27 nonfatigued). Resting-state EEGs were analyzed, and associations with fatigue ratings and group differences were assessed. Logistic mixed-effects regression models evaluated classification accuracy for fatigue status.

**Results:**

Lower frontal aperiodic exponents were associated with higher cognitive fatigue across participants. Fatigued individuals, regardless of diagnosis, showed reduced frontal exponent values compared with nonfatigued groups, while no differences emerged in occipital regions. Logistic regression confirmed that frontal exponent values significantly predicted fatigue status, improving classification accuracy beyond age and depression, with good sensitivity and specificity.

**Conclusions:**

The frontal aperiodic exponent is a regionally specific biomarker of cognitive fatigue across MS and LCOF. Mechanistic interpretation suggests an altered prefrontal E/I balance, which could inform the development of targeted interventions to alleviate cognitive fatigue. It offers a clinically accessible tool to complement self-report, support trial stratification, and enable objective treatment monitoring. Importantly, its presence across distinct disorders highlights its value as a transdiagnostic marker of fatigue.

## Introduction

Fatigue is a complex and disabling symptom, marked by persistent exhaustion that typically worsens throughout the day and significantly interferes with daily functioning (Beatty et al., 2003). It is prevalent across various neurological conditions, with particularly high incidence in multiple sclerosis (MS), where it affects up to 80% of patients and is often cited as one of the most debilitating symptoms (Oliva Ramirez et al., 2021). Similarly, fatigue is one of the most commonly reported and disabling symptoms in Long COVID. Long COVID is generally defined as the continuation or emergence of new symptoms 3 months postinfection with SARS-CoV-2, lasting for at least 2 months and not attributable to alternative diagnoses (WHO, 2025). As in MS, individuals with Long COVID-related fatigue (LCOF) frequently describe fatigue as a major factor reducing their quality of life, with substantial personal and socioeconomic implications (Ceban et al., 2022). Notably, many individuals report prominent cognitive fatigue, characterized by mental exhaustion, impaired concentration, and reduced cognitive performance, which contributes to the overall burden of the symptom.

Despite extensive research, the pathophysiological mechanisms underlying cognitive fatigue remain elusive, largely due to its subjective and often invisible nature, which complicates reliable assessment. A wide range of studies has aimed to supplement self-reported fatigue by examining objective indicators, including both behavioral markers (such as reaction time variability and accuracy) and electrophysiological measures (see Linnhoff et al. [2019] for a comprehensive overview). However, a consistent challenge across these approaches is their limited correlation with subjective fatigue ratings, making it difficult to quantify fatigue severity reliably. Moreover, many of these studies have focused exclusively on fatigued individuals, with limited inclusion of individuals with MS without fatigue as controls. In the context of LCOF, research into objective markers is even more limited and still in its early stages.

Recent findings shed new light on the neurochemical mechanisms underlying cognitive fatigue by demonstrating that prolonged cognitive effort leads to glutamate accumulation in the left lateral prefrontal cortex, potentially impairing cortical efficiency (Wiehler et al., 2022). Glutamate plays a central role in the regulation of the excitation/inhibition (E/I) balance, which reflects the interplay between glutamate-driven excitation and Gamma-Aminobutyric Acid (GABA)-mediated inhibition and is crucial for stable neural function. This balance is typically quantified in vivo using neuroimaging approaches, such as magnetic resonance spectroscopy (MRS) (Finkelman, Furman-Haran, Paz, & Tal, 2022). Disruptions in this balance are implicated in various neurological and psychiatric disorders, including MS (He & Cline, 2019).

Excessive levels of glutamate, particularly in the extracellular space, are known to be potentially toxic for neural functions and can lead to neuronal damage (Macrez et al., 2016; Olney & Sharpe, 1969; Rajda et al., 2017). This so-called glutamate excitotoxicity is thought to contribute significantly to the progression of the MS disease (Kostic, Zivkovic, & Stojanovic, 2013; Macrez et al., 2016; Rajda et al., 2017). Although a direct link between glutamate and MS-related fatigue has not yet been established, symptom-alleviating effects of glutamate antagonists, such as amantadine, suggest a possible involvement of glutamatergic mechanisms in the fatigue development (Macrez et al., 2016).

While MRS provides a valuable noninvasive measure of regional neurotransmitter concentrations, it primarily reflects a more stable neurochemical tone, including nonsynaptic pools, such as metabolic or glial glutamate, and is therefore less suited for capturing rapid, ongoing neural dynamics. In contrast, EEG offers a noninvasive means to assess functional aspects of neural activity, including the E/I balance, particularly through the analysis of aperiodic activity (Cohen Kadosh, 2025). Thus, in recent years, EEG analysis has expanded beyond the traditional focus on oscillatory activity to include aperiodic components, which were long dismissed as background noise. Emerging evidence indicates that the aperiodic component carries physiologically meaningful information and may serve as a marker of the E/I balance (Donoghue et al., 2020; Gao, Peterson, & Voytek, 2017; Lu, Dermody, Duncan, & Woolgar, 2024).

The E/I balance can be approximated via the aperiodic exponent of the EEG power spectrum in log–log space. This exponent, denoted as “*x*” in the 1/*f^x^* function, reflects the relative contributions of excitation and inhibition: steeper (more negative) slopes suggest increased inhibitory (GABAergic) activity, whereas flatter slopes indicate a shift toward excitatory (glutamatergic) activity (Donoghue et al., 2020; Gao et al., 2017; Gerster et al., 2022).

To date, few studies have investigated the aperiodic exponent in fatigue, and even fewer have adopted a transdiagnostic perspective encompassing both MS and LCOF. Understanding shared neurophysiological mechanisms across conditions could inform the development of targeted interventions and objective monitoring tools. Accordingly, the present study examined the aperiodic exponent as an EEG-based proxy of the E/I balance in individuals with MS and LCOF, as well as healthy controls (HCs). We hypothesized that the aperiodic exponent would differ systematically between fatigued and nonfatigued individuals, and that higher levels of fatigue would be associated with flatter slopes, indicative of a relative shift toward cortical excitation.

## Materials and methods

### Study sample

Resting-state EEG recordings were collected from HCs and from individuals with MS and LCOF between 2022 and 2024. All recordings were acquired using an identical EEG setup to ensure consistency. Participants were recruited as a consecutive convenience sample, and group allocation was based solely on the predefined inclusion and exclusion criteria; no randomization was performed. Resting-state EEGs were recorded before any experimental tasks or interventions. A total of 119 participants were included in the final sample. They were divided into three groups: 36 HCs (HC group), 33 individuals with LCOF (LCOF group), and 50 individuals with MS (MS group) (see Table 1 for demographic and clinical characteristics). Inclusion criteria for the HCs required the absence of any history of neurological or psychiatric disorders and no current symptoms of depression, as determined by a score ≤ 4 on the Beck Depression Inventory II – Fast Screen (BDI-FS). Individuals with MS were included if they had a clinically definite diagnosis of MS according to the McDonald criteria (Thompson et al., 2018) and were at least 3 months post-relapse and corticosteroid treatment. Additional inclusion criteria for the MS group included the absence of current neurological or psychiatric comorbidities and no current use of fatigue or antidepressant medications. All continued their usual disease-modifying treatments. Among the 50 individuals with MS, 48 had a relapsing–remitting course, 1 had a secondary-progressive course, and 1 had a primary-progressive course. Fatigue severity was assessed using the Wuerzburg Fatigue Inventory for Multiple Sclerosis (WEIMuS) or the Modified Fatigue Impact Scale (MFIS). Using established cutoff scores for the cognitive subscales of MFIS (>18; Fisk et al., 1994) and WEIMuS (>17; Flachenecker et al., 2006), participants in the MS group were stratified into fatigued (MS + F, *n* = 23) and nonfatigued (MS − F, *n* = 27) subgroups. Subjective fatigue ratings were missing for 26 HCs. Analyses involving these scores, therefore, included only participants with available data. Inclusion criteria for the individuals with LCOF required a confirmed prior infection with SARS-CoV-2 and a cognitive fatigue score exceeding the clinical cutoff of 17 on the WEIMuS (Flachenecker et al., 2006). Individuals with LCOF were excluded if they presented with acute psychiatric or neurological conditions or were taking psychotropic medications at the time of testing. The study was approved by the local ethics committee of the University of Magdeburg, and all participants provided written informed consent in accordance with the Declaration of Helsinki.

**Table 1.** Group characteristics, mean (± SD)

			MS		
	HC	LCOF	MS + F	MS − F	
Gender (f/m)	26/10	27/6	18/5	18/9	*p* = .560
Age (years)	36.42 (± 14.19)	45.49 (± 11.71)	41.04 (± 10.07)	40.29 (± 13.25)	*p* = .038
BDI-FS (points)	1.17 (± 1.23)	2.58 (± 1.95)	4.00 (± 2.88)	2.00 (± 2.00)	*p* < .001
Fatigue score (*z*-score)	−1.09 (± 0.85)	1.04 (± 0.55)	0.86 (± 0.48)	−0.96 (± 0.65)	*p* < .001
Disease duration (years)			8.73 (± 6.96)	9.82 (± 9.22)	*p* = .640
EDSS (points)			2.85 (± 1.58)	2.09 (± 1.26)	*p* = .073

*Note:* Group differences were assessed using *χ^2^* tests for categorical variables (gender), one-way ANOVA for age, BDI-FS, and cognitive fatigue scores, and independent-samples *t*-tests for disease duration and EDSS.

BDI-FS, becks depression inventory – fast screen; EDSS, expanded disability status scale; HC, healthy controls; LCOF, Long-COVID-related fatigue; MS, multiple sclerosis (+F, with fatigue, −F, without fatigue).

### Procedure

All individuals provided written informed consent before participation and completed standardized assessments of depressive symptoms (BDI-FS) and subjective fatigue (MFIS or WEIMuS). EEG recording durations ranged from 4 to 8 min, and for all participants, at least 3 min of the eyes-closed condition were available and subsequently analyzed.

### EEG signal recording and preprocessing

The continuous EEG signal was recorded from 14 scalp electrodes (F3, Fz, F4, FCz, C3, Cz, C4, P3, Pz, P4, POz, O1, Oz, and O2), using Ag/AgCl electrodes mounted in an elastic cap (EasyCap GmbH, Germany). The ground electrode was placed at AFz, and all EEG channels were offline re-referenced to the averaged signal of the left and right mastoids. Additionally, an electrooculogram was recorded to monitor eye movements. EEG signals were acquired using a BrainAmp DC amplifier (Brain Products, Germany), with a sampling rate of 1,000 Hz. Electrode impedances were kept below 5 kΩ.

Preprocessing of the resting-state EEG data was conducted in MATLAB (R2024b) using the FieldTrip toolbox (Oostenveld, Fries, Maris, & Schoffelen, 2011). Three-minute eyes-closed resting-state EEG segments were analyzed in line with previous work employing the Fitting Oscillations and One Over F (FOOOF) toolbox to estimate the aperiodic exponent (Arnett et al., 2024; Donoghue et al., 2020). Data were high-pass filtered at 0.5 Hz, low-pass filtered at 100 Hz, and notch filtered at 50 Hz. The data were then segmented into nonoverlapping epochs of 1-s duration. Spectral analysis was performed using the multitaper method with power spectra estimated across the 1–90 Hz frequency range. The power spectral density was subsequently parameterized using the FOOOF toolbox (version 20250114; Donoghue et al., 2020), which separates aperiodic and periodic components of the EEG signal. The primary feature of interest was the aperiodic slope exponent, defined as the steepness of the power spectrum in log–log space. This measure is considered an index of the cortical E/I balance, with steeper slopes indicating greater inhibitory activity and flatter slopes reflecting a shift toward excitatory activity.

### Statistical analysis

**Figure 1.**
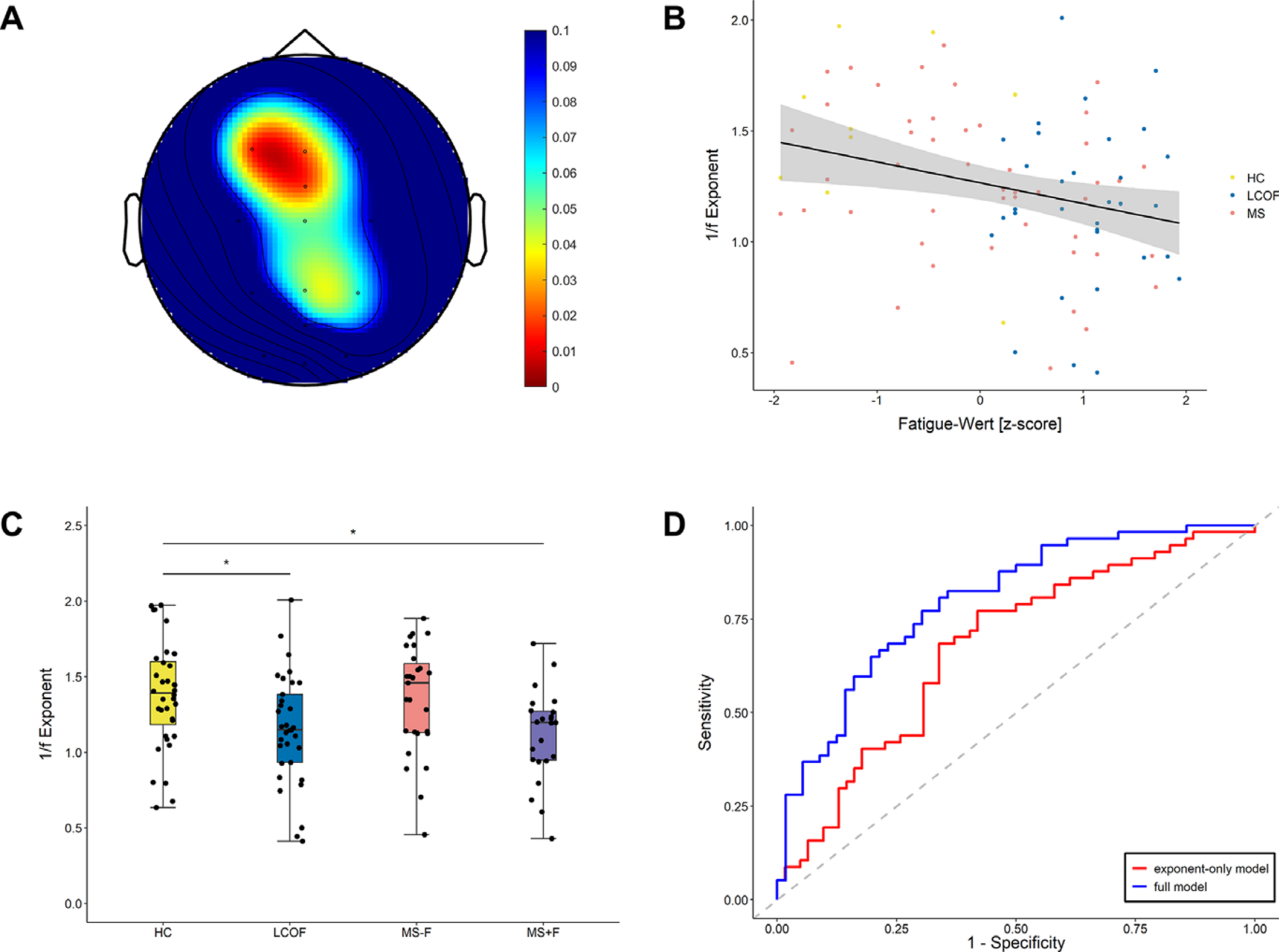
Multifaceted evidence linking frontal aperiodic exponent to fatigue. (a) Topographical distribution of *p*-values from Kendall’s 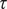 correlations between aperiodic exponent values and subjective fatigue scores. Electrodes are color-coded according to the statistical significance of the observed correlations. (b) Group comparison of the aperiodic exponent values in the frontal region of interest. Boxplots show average aperiodic exponent values for each group (HCs, LCOF, and MS with fatigue [MS + F] and without fatigue [MS − F]), with individual data points overlaid (*p < .05). (c) Association between subjective fatigue scores and the average aperiodic exponent values within the frontal region of interest. Each point represents a single subject, color-coded by group (HC, LCOF, and MS group). The black line indicates the linear trend with a 95% confidence interval. (d) Receiver operating characteristic (ROC) curves for the full logistic regression model (blue) and the model using only the aperiodic exponent as a predictor (red). The dashed diagonal line represents the performance of a random classifier.

All statistical analyses and visualizations were conducted using R (version 4.2.0; R Core Team) and MATLAB. Outliers were identified as values exceeding 1.5 times the interquartile range and were winsorized to reduce their influence without excluding any data points. Fatigue scores from the MFIS and WEIMuS cognitive subscales were standardized (*z*-scores) to allow for comparability across instruments. Electrode-wise correlations between aperiodic exponent values and cognitive fatigue scores were first computed using Kendall’s *τ* to explore the spatial distribution of effects (see Figure 1a). Based on these maps, a frontal region of interest (ROI) was defined, comprising electrodes F3, Fz, and FCz. Associations between mean frontal ROI exponent values and fatigue scores were assessed using Kendall’s rank correlation coefficients. Next, group differences in mean aperiodic exponent values across the defined ROI were examined using one-way analysis of variance (ANOVA) followed by Tukey’s honest significant difference (HSD) post-hoc tests. Finally, to evaluate the diagnostic utility of the aperiodic exponent, binary logistic mixed-effects regression models were constructed to predict fatigue status (0 = nonfatigued, 1 = fatigued) based on established cutoff scores of MFIS (>18; Fisk et al., 1994) or WEIMuS (>17; Flachenecker et al., 2006) cognitive subscales. The full model included frontal ROI exponent values as the main predictor, with age and depression scores as covariates, and participants as random intercepts. Model performance was evaluated via sensitivity, specificity, and area under the receiver operating characteristic (ROC) curve (AUC). Model comparison was conducted using *χ* ^2^ tests, Akaike’s information criterion (AIC) values, and changes in pseudo-*R* ^2^ to quantify the incremental predictive value of the aperiodic exponent relative to covariates.

## Results

### Correlational analysis between the aperiodic exponent and fatigue

Within the predefined frontal ROI, a significant negative association was observed between mean aperiodic exponent values and subjective fatigue scores across all participants (Kendall’s 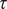 = −0.17, *p* = .015, *n* = 93). Thus, this finding indicates a graded relationship between fatigue severity and cortical excitability, with higher fatigue levels associated with lower (flatter) aperiodic exponent values over frontal electrodes (see Figure 1b).

### Group differences in aperiodic exponent

To investigate group differences in aperiodic exponent values within the frontal ROI, a one-way ANOVA was conducted. The assumption of normality was confirmed via the Shapiro–Wilk test (*p* > .05). The ANOVA revealed a significant main effect of group on the aperiodic exponent values (*F*(3, 115) = 4.29, *p* = .007, 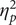 = 0.102, Figure 1c). Post-hoc pairwise comparisons, using Tukey’s HSD test to control for family-wise error, indicated that participants in the MS + F group exhibited significantly lower aperiodic exponent values compared to the HC group (adjusted *p* = .028). Similarly, aperiodic exponent values in the LCOF group were significantly lower than in the HC group (adjusted *p* = .045). No other between-group differences reached statistical significance (all *p*s > .09).

To directly test our primary hypothesis of a transdiagnostic fatigue effect, a planned contrast was performed comparing all fatigued participants (MS + F and LCOF group) against the HC group, as well as a pooled nonfatigued group (HC and MS-F group). Frontal aperiodic exponents were significantly lower in fatigued participants than in both the HC (*t*(115) = −3.24, *p* = .002, Cohen’s *d* = −0.70) and the nonfatigue group (*t*(115) = −3.52, *p* < .001, Cohen’s *d* = −0.66).

To assess regional specificity, a supplementary analysis was conducted on the aperiodic exponent values in an occipital ROI (POz, Oz, and O1). Due to violations of the normality assumption, a Kruskal–Wallis test was employed. No significant group differences were observed (*χ^2^*(3) = 1.64, *p* = .65), further supporting the topographical specificity of the frontal effect (see Figure S1 in the Supplementary Material).

### Predictive utility of the aperiodic exponent

To assess the diagnostic utility of the aperiodic exponent in classifying fatigue status, a binary logistic mixed-effects regression model was fitted with the aperiodic exponent values from the frontal ROI, age, and depression scores (BDI-FS) as fixed effects, and participants as random intercepts. The analysis revealed that frontal exponent values were a significant predictor of fatigue status (*β* = −2.170, *z* = −2.717, *p* = .006), indicating that lower aperiodic exponent values are associated with a higher likelihood of belonging to the fatigued group. Depression scores were a significant predictor (*β* = 0.425, *z* = 2.972, *p* = .003), suggesting that higher depression scores also increase the likelihood of belonging to the fatigued group. Age, however, was not a significant predictor in the model (*β* = 0.027, *z* = 1.390, *p* = .16).

Model comparison indicated that the full model (aperiodic exponent, age, and depression) provided a significantly better fit than a reduced model, including only age and depression (*χ^2^* (2) = 11.63, *p* < .001). The improvement is further supported by a decrease in AIC from 143.28 (reduced model) to 133.66 (full model) and an increase in explained variance, with pseudo-*R* ^2^ rising from 24.79% to 36.2%. These findings confirm the incremental predictive value of the aperiodic exponent for fatigue classification.

ROC analysis showed that the achieved AUC for the full model was 0.78, indicating good classification performance. The model showed a sensitivity of 71% and a specificity of 74%, indicating balanced predictive accuracy. Additionally, we evaluated the predictive performance of a model including only the aperiodic exponent to assess its standalone discriminatory power. The exponent-only model yielded an AUC of 0.68, with a sensitivity of 58% and a specificity of 69%, suggesting that the aperiodic exponent alone provides moderate discriminatory power. Figure 1d displays the ROC curves for both models.

## Discussion

This study investigated the relationship between cortical E/I (im-)balance, quantified via the aperiodic exponent of the EEG power spectrum, and subjective fatigue in individuals with MS and LCOF, as well as HCs. Based on the theoretical premise that E/I balance is critical for cognitive regulation, we hypothesized that flatter aperiodic slopes, which suggest higher E/I, would be associated with higher levels of subjective fatigue.

The results provided strong support for this hypothesis. A significant negative correlation was observed between the aperiodic exponent values and subjective fatigue scores, particularly over frontal electrode sites. Individuals with higher levels of fatigue exhibited flatter slopes, consistent with increased excitatory influence in frontal cortical regions. The strongest correlation emerged over the left dorsolateral prefrontal cortex (dlPFC), a region known to be centrally involved in cognitive control and previously implicated in fatigue-related mechanisms (Ayache & Chalah, 2017). Group comparisons further confirmed these findings: both individuals with MS and fatigue (MS + F group) and individuals with LCOF (LCOF group) showed significantly flatter slopes compared to HCs, further supporting the interpretation of a disrupted E/I balance in fatigued individuals. Notably, no significant differences were observed over occipital electrodes, underscoring the spatial specificity of the effect and reinforcing the functional relevance of frontal cortical areas in the neural signature of fatigue.

The dlPFC plays a central role in several cognitive functions, such as sustained attention and cognitive control (Clayton, Yeung, & Cohen Kadosh, 2015; Friedman & Robbins, 2022). A well-established oscillatory correlate of prolonged cognitive engagement is frontomedial theta power (Clayton et al., 2015), which reliably increases during tasks requiring sustained attention (Boksem, Meijman, & Lorist, 2005; Craig, Tran, Wijesuriya, & Nguyen, 2012; Linnhoff, Wolter-Weging, & Zaehle, 2021; Wascher et al., 2014). This increase is thought to reflect the upregulation of cognitive control mechanisms, supporting goal maintenance and performance monitoring under conditions of mental effort (Clayton et al., 2015). In the context of sustained mental effort and emerging fatigue, this frontomedial theta power enhancement is thought to act as a compensatory mechanism that helps sustain cognitive performance during active task engagement.

However, our study examines resting-state EEG, where different neural signatures of fatigue may emerge. One possible mechanistic link is that the compensatory theta increase during active tasks reflects an initial upregulation of cognitive control that relies on increased excitatory drive. Over time, prolonged activation of the cognitive control network, particularly within prefrontal regions, is likely associated with increased glutamate release, as suggested by the findings of Wiehler et al. (2022). Persistent glutamate accumulation in the prefrontal cortex, especially under high cognitive load, may exceed homeostatic limits, resulting in disrupted cortical efficiency and impaired cognitive performance (Macrez et al., 2016; Olney & Sharpe, 1969; Rajda et al., 2017). This shift can manifest in the resting state as a flattening of the aperiodic exponent, reflecting broadband spectral changes independent of oscillatory power modulations (Donoghue et al., 2020).

Such a framework reconciles the coexistence of increased frontomedial theta during active task performance and flatter aperiodic slopes at rest: both may reflect different stages or aspects of fatigue-related neural dynamics, with the former representing an acute, task-related compensatory process, and the latter a downstream consequence of altered neurochemical balance. This distinction between active and resting-state conditions may help explain discrepancies with findings in other contexts (e.g., Attention-Deficit/Hyperactivity Disorder (ADHD) and transcranial random noise stimulation (tRNS) interventions) and offers a testable hypothesis for future research.

Supporting this interpretation, Lu et al. (2024) showed that aperiodic broadband activity reliably tracks cognitive demand across multiple tasks in healthy individuals. While their study captured transient, task-related shifts in aperiodic power, our findings demonstrate that individuals with fatigue, including those with MS and LCOF, exhibit similar aperiodic changes, specifically flatter slopes in prefrontal regions, which mark a more persistent, potentially maladaptive cortical state in individuals experiencing chronic fatigue. This electrophysiological signature may reflect glutamatergic overload, as hypothesized to impair the efficiency of cognitive control processes. Importantly, our results complement previous observations showing that fatigued individuals with MS fail to exhibit the typical compensatory increase in frontomedial theta power during prolonged cognitively demanding tasks (Linnhoff, Haghikia, & Zaehle, 2023), potentially due to chronically elevated glutamate levels that limit adaptive neural recruitment. Furthermore, whereas healthy individuals appear to benefit from breaks that support glutamate clearance and restore prefrontal function (Wiehler et al., 2022), this recovery mechanism may be compromised in individuals with MS fatigue and LCOF (Dobryakova, Deluca, Genova, & Wylie, 2013; Leocani, Colombo, & Comi, 2008). Such compromised neurochemical recovery could contribute to the persistence of fatigue and the associated decline in cognitive performance, even in the absence of ongoing task demands.

Recent work indicates that fatigue is accompanied by alterations across multiple levels of neural organization. EEG microstate studies show that fatigued individuals exhibit changes in large-scale temporal network dynamics, including reduced expression of microstate F and increased expression of microstate B in MS (Baldini et al., 2023; Baldini et al., 2024), as well as systematic shifts in the occurrence and coverage of microstates C and D during experimentally induced mental or physical (state) fatigue (Li, Cheng, Wang, & Chang, 2023; Zhao, Lin, Chi, & Gao, 2023). Complementary EEG-based connectivity studies further demonstrate that (trait) fatigue is associated with characteristic reorganization of functional networks in post-stroke fatigue (Wu, Doncker, & Kuppuswamy, 2023), and consistent patterns of reduced fronto-parietal and increased posterior or sensorimotor coupling during sustained cognitive load, driving (state) fatigue, or physical exertion (Chuang et al., 2018; Sun et al., 2014; Wang et al., 2016). Within this multidimensional context, the aperiodic exponent provides a mechanistically distinct signal: rather than reflecting network-level coordination, it might index the underlying balance between cortical excitation and inhibition, capturing a more fundamental property of neuronal population activity. Our finding that the aperiodic exponent predicts trait fatigue, therefore, suggests that fatigue may not only involve altered large-scale network dynamics but also shifts in cortical excitation–inhibition balance, positioning the aperiodic exponent as a complementary neurophysiological marker within the broader spectrum of EEG-based fatigue indicators.

The predictive modeling results further support the relevance of the aperiodic exponent as a potential biomarker for fatigue. The logistic regression model confirmed that the exponent values significantly predicted group membership, with individuals exhibiting flatter slopes being more likely to belong to the fatigued group. Importantly, these findings highlight the potential of EEG-derived biomarkers to distinguish fatigued from nonfatigued individuals independently of subjective self-reports. However, while the exponent successfully differentiated between fatigued and nonfatigued groups, the model’s moderate sensitivity and specificity suggest that it may not yet be sufficient as a standalone diagnostic tool. However, direct comparison of our AUC of 0.78 with previous EEG-based fatigue markers is limited by the scarcity of studies reporting ROC metrics. Available work has focused almost exclusively on state or driving-related fatigue, with reported AUC values between ~0.90 and 0.97 (Hu, 2017; Subasi et al., 2022), while many other EEG studies report only accuracies without ROC analyses (F. Wang et al., 2023). As AUC cannot be inferred from accuracy and our model targets the trait rather than state fatigue, these values are not directly comparable. Furthermore, studies on fatigue-related alterations in EEG microstates or connectivity do not provide AUC estimates, further constraining cross-study comparisons.

The aperiodic exponent should be regarded as a promising adjunctive marker, one that complements, but does not replace, established subjective assessments of fatigue in clinical practice. As expected, depressive symptomatology also emerged as a significant predictor of fatigue status, reflecting well-documented overlap between fatigue and mood-related symptoms, such as reduced motivation, impaired concentration, and diminished energy (Bakshi et al., 2000). Despite this overlap, our findings demonstrated that the aperiodic exponent explained additional variance in fatigue beyond age and depression scores, highlighting its value as a distinct neurophysiological marker of fatigue.

Beyond its diagnostic relevance, the identification of a shared electrophysiological signature of fatigue in both individuals with MS fatigue and LCOF also carries important transdiagnostic implications. The presence of reduced aperiodic exponent values over frontal electrodes, across two clinically distinct conditions, suggests that similar neurophysiological alterations may underlie fatigue regardless of etiology. Notably, similar aperiodic power changes have been observed in healthy individuals during cognitively demanding tasks (Lu et al., 2024). Together, these findings emphasize the value of aperiodic EEG marker as a transdiagnostic indicator of cognitive dysregulation, reflecting both transient task-related dynamics and more persistent pathological states. This convergence points to the dlPFC as a potential common hub of vulnerability in fatigue, possibly reflecting a final common pathway of disrupted cognitive control due to impaired E/I balance.

These findings raise the possibility that fatigue, while emerging in the context of different diseases, may be driven by overlapping cortical mechanisms, a perspective that could inform more generalizable intervention strategies across patient populations.

While our findings provide promising evidence for the use of the aperiodic exponent as an objective marker of fatigue, some limitations must be acknowledged. First, the cross-sectional design of this study precludes any inference regarding causality. Future longitudinal studies, or interventional studies, are needed to provide a causal inference of the role of E/I balance in fatigue or whether fatigue contributes to progressive changes in excitatory-inhibitory dynamics.

Second, our interpretation of flatter slopes as reflecting increased glutamatergic activity remains indirect and speculative. The present study relied exclusively on EEG data and did not include direct measures of neurotransmitter concentrations. While our findings are consistent with prior reports of glutamate accumulation in prefrontal regions during cognitive effort (Wiehler et al., 2022), it is important to acknowledge that EEG and MRS likely capture distinct but complementary aspects of cortical excitation and inhibition. EEG may reflect dynamic, state-dependent changes in neuronal excitability, whereas MRS provides a more stable neurochemical profile (Cohen Kadosh, 2025). Future multimodal studies integrating both approaches are needed to more precisely characterize the relationship between electrophysiological and neurochemical indicators of E/I balance.

Third, the present study focused solely on resting-state EEG. Although resting-state measures provide valuable insights into baseline cortical dynamics, they may not capture task-related modulations of the E/I balance that occur in response to mental effort. Incorporating task-based EEG paradigms in future research could elucidate whether the observed aperiodic exponent alterations persist, intensify, or adapt during cognitive challenge. Such data would offer a more dynamic understanding of how fatigue manifests in both resting and active brain states and may help to delineate compensatory versus pathological neural processes.

In conclusion, our findings demonstrate that the frontal aperiodic exponent is a robust, regionally specific, and clinically relevant correlate of cognitive fatigue across neurological and post-viral conditions. By identifying a shared neurophysiological signature in both MS-related fatigue and LCOF, this study provides evidence for a transdiagnostic mechanism underlying fatigue, independent of disease etiology. Importantly, the aperiodic exponent showed incremental predictive value for classifying fatigue status beyond age and depressive symptoms, highlighting its potential utility as an objective EEG-based biomarker. These results not only advance mechanistic understanding of fatigue in clinical populations but also pave the way for the development of targeted interventions and objective monitoring tools, supporting more precise diagnosis, stratification, and treatment evaluation in both research and clinical practice.

## Supporting information

10.1017/S0033291725103024.sm001 Linnhoff et al. supplementary material Linnhoff et al. supplementary material

## Supplementary material

The supplementary material for this article can be found at http://doi.org/10.1017/S0033291725103024.
